# Gaussian-binary restricted Boltzmann machines for modeling natural image statistics

**DOI:** 10.1371/journal.pone.0171015

**Published:** 2017-02-02

**Authors:** Jan Melchior, Nan Wang, Laurenz Wiskott

**Affiliations:** Institut für Neuroinformatik, Ruhr-Universität Bochum, D-44801 Bochum, Germany; University of Bonn, Bonn-Aachen International Center for IT, GERMANY

## Abstract

We present a theoretical analysis of Gaussian-binary restricted Boltzmann machines (GRBMs) from the perspective of density models. The key aspect of this analysis is to show that GRBMs can be formulated as a constrained mixture of Gaussians, which gives a much better insight into the model’s capabilities and limitations. We further show that GRBMs are capable of learning meaningful features without using a regularization term and that the results are comparable to those of independent component analysis. This is illustrated for both a two-dimensional blind source separation task and for modeling natural image patches. Our findings exemplify that reported difficulties in training GRBMs are due to the failure of the training algorithm rather than the model itself. Based on our analysis we derive a better training setup and show empirically that it leads to faster and more robust training of GRBMs. Finally, we compare different sampling algorithms for training GRBMs and show that Contrastive Divergence performs better than training methods that use a persistent Markov chain.

## Introduction

Inspired by the hierarchical structure of the visual cortex, recent studies on probabilistic models have used deep hierarchical architectures to learn higher order statistics of image data [1–3]. One widely used architecture is a stack of restricted Boltzmann machines (RBMs) known as a deep belief network [4–6]. Since the original formulation of RBMs assumes binary input values, the model needs to be modified in order to handle continuous input values. One common way is to replace the binary input units by linear units with independent Gaussian-noise. The resulting model is known as Gaussian-binary restricted Boltzmann machines (GRBMs) or Gaussian-Bernoulli restricted Boltzmann machines [7–9].

The difficulties of training GRBMs, in particular for modeling natural images, have been reported by several authors [8–19] and various modifications have been proposed to address this problem. Lee et al. [10] have used a sparse penalty during training of GRBMs, which allowed them to learn meaningful features from natural image patches. Krizhevsky [8] has trained GRBMs on natural images and has hypothesized that the difficulties are mainly due to high-frequency noise in the images, which prevents the model from learning the important structures. Ranzato and Hinton [13, 14] have argued that the failure of GRBMs is due to the model’s limitations in modeling covariances and have proposed an advanced model that explicitly models covariances. Nair et al. [12] and Courville et al. [15] have suggested that the poor ability of GRBMs in modeling natural image statistics is mainly due to the binary nature of the hidden units and proposed advanced models with real-valued hidden units. Theis et al. [11] have illustrated that in terms of likelihood estimation, GRBMs are outperformed even by simple mixture models. Cho et al. [9] have suggested that the failure of GRBMs is due to the training algorithm and have proposed a modified sampling algorithm and an adaptive learning rate.

The studies above have shown the failures of GRBMs empirically. However, to our knowledge there is no formal analysis of GRBMs that investigates the reasons behind these failures, apart from our preliminary work [17–19]. In this paper, we extend our work on considering GRBMs from the perspective of density models, i.e. how well the model learns the distribution of the data. We show that a GRBM can be regarded as a mixture of Gaussians (MoG), which has already been mentioned briefly in previous studies [11, 15, 20] but has gone unheeded. Furthermore, in the case of binary visible and binary hidden variables, the relationship of an RBM and a mixture model has originally been shown by Freund et al. [21]. In this paper, we show that a GRBM is in fact a constrained MoG, where the Gaussian components cannot be placed independently of each other, and how this limits the way the model can represent the data. We argue, however, that due to the exponential number of components it does not prevent the model from learning the statistical structure in the data. We present successful training of GRBMs both on a two-dimensional blind source separation problem and on natural image patches without using additional regularization methods such as a sparse penalty. The results are comparable to those of independent component analysis (ICA), which is generally accepted to be a good model for natural image statistics. We compare different sampling algorithms for training GRBMs and show that Contrastive Divergence performs better than training methods that use a persistent Markov chain. Based on our analysis, we derive a better initialization for the model parameters and propose to restrict the gradient to a reasonable size. We illustrate empirically how these modifications in combination with Contrastive Divergence help to overcome the reported problems and lead to fast and robust training of GRBMs. Finally, we discuss the advantages and limitations of GRBMs in comparison to other generative models.

## Gaussian-binary restricted Boltzmann machines

### The model

A Boltzmann Machine (BM) is a Markov Random Field with stochastic *visible* and *hidden* units [22], which are denoted as **X** ≔ (*X*_1_, …, *X*_*M*_)^*T*^ and **H** ≔ (*H*_1_, …, *H*_*N*_)^*T*^, respectively. In general, we use bold letters to denote vectors and matrices. The joint probability distribution is defined as

$$P\left(X,H\right)≔\frac{1}{Z}e^{-\frac{1}{T_{0}}E\left(X,H\right)},$$ (1)

$$Z≔\int \int e^{-\frac{1}{T_{0}}E\left(x,h\right)}dx\;dh$$ (2)

where *E*(**X**, **H**) denotes an *energy function* as known from statistical physics, which defines the dependence between **X** and **H**. The temperature parameter *T*_0_ is usually ignored by setting its value to one, but it can play an important role in inference of BMs [23]. The *partition function* *Z* normalizes the probability distribution by integrating over all possible values of **X** and **H**, which is intractable in most cases, such that in training BMs using gradient descent the partition function is usually estimated using sampling methods. However, even sampling in BMs is difficult due to the dependencies between all variables.

An RBM is a special case of a BM where the energy function contains no terms combining two different hidden or two different visible units. Viewed as a graphical model, there are no lateral connections within the visible or hidden layer, which results in a bipartite graph. This implies that the hidden units are conditionally independent given the visibles and *vice versa*, which allows efficient sampling.

The values of the visible and hidden units are usually assumed to be binary, i.e. *X*_*m*_, *H*_*n*_ ∈ {0, 1}. The most common way to extend an RBM to continuous data is a GRBM, which assumes continuous values for the visible units and binary values for the hidden units. Its energy function [9, 17] is defined as

$$E\left(X,H\right):\;=\sum_{i}^{M}\frac{{\left(X_{i}-b_{i}\right)}^{2}}{2\sigma^{2}}-\sum_{j}^{N}c_{j}H_{j}-\sum_{i,j}^{M,N}\frac{X_{i}w_{ij}H_{j}}{\sigma^{2}}$$ (3)

$$=\frac{∥X-{b∥}^{2}}{2\sigma^{2}}-c^{T}H-\frac{X^{T}WH}{\sigma^{2}},$$ (4)

where ∥**u**∥ denotes the Euclidean norm of **u**. The conditional probability distributions are given by

$$P\left(H_{j}=1|x\right)\overset{\left(1,2,4\right)}{=}\frac{1}{1+e^{-c_{j}-\frac{x^{T}w_{*j}}{\sigma^{2}}}},$$ (5)

$$P\left(X_{i}|h\right)\overset{\left(1,2,4\right)}{=}N\left(X_{i};b_{i}+w_{i*}^{T}h,\sigma^{2}\right),$$ (6)

where **w**_i*_ and w_*j_ denote the *i*th row and the *j*th column of the weight-matrix, respectively. $N(x;\mu ,\sigma^{2})$ denotes a Gaussian distribution with mean *μ* and variance *σ*^2^. For a detailed derivation of the conditional distributions see Wang et al. [24]. To keep the notation in our analysis simple we use the same standard deviation in all dimensions (see Melchior [18] for an analysis with independent standard deviations).

### Maximum likelihood estimation

Maximum likelihood estimation (MLE) is a frequently used technique for training probabilistic models like BMs. In MLE we have a data set $\tilde{X}=\{{\tilde{x}}_{1},\ldots ,{\tilde{x}}_{L}\}$ where the observations ${\tilde{x}}_{l}$ are presumed to be independent and identically distributed (i.i.d.). The goal is to find optimal parameters $\tilde{\Theta }$ that maximize the likelihood of the data, i.e. maximize the probability that the data could be generated by the model [25]. For practical reasons one often considers the logarithm of the likelihood, which has the same maximum as the likelihood since the logarithm is a strictly monotonic function. The log-likelihood is defined as

$$\ln P\left(\tilde{X};\Theta \right)=\ln\prod_{l=1}^{L}P\left({\tilde{x}}_{l};\Theta \right)=\sum_{l=1}^{L}\ln P\left({\tilde{x}}_{l};\Theta \right).$$ (7)

We use the average log-likelihood per training sample denoted by $\hat{\ell }$. For RBMs it is defined as

$$\hat{\ell }≔{\langle \ln P\left(\tilde{X};\Theta \right)\rangle }_{\tilde{x}}={\langle \ln\left(\sum_{h}e^{-E\left(\tilde{x},h\right)}\right)\rangle }_{\tilde{x}}-\ln Z,$$ (8)

where $\tilde{x}\in \tilde{X}$, and 〈*f*(*u*)〉_*u*_ denotes the expectation value of the function *f*(*u*) with respect to variable *u*, which in this case is just the average due to the i.i.d. assumption of $\tilde{x}$. The gradient of $\hat{\ell }$ turns out to be the difference between the expectation values of the energy gradient under the data and model distribution, which is given by

$$\frac{\partial \hat{\ell }}{\partial \theta }\;\overset{\left(1,2,8\right)}{=}\;-{\langle \sum_{h}P\left(h|\tilde{x}\right)\frac{\partial E\left(\tilde{x},h\right)}{\partial \theta }\rangle }_{\tilde{x}}+{\langle \sum_{h}P\left(h|x\right)\frac{\partial E\left(x,h\right)}{\partial \theta }\rangle }_{x}.$$ (9)

See [18, 26] for a detailed derivation. In practice, a finite set of i.i.d. samples can be used to approximate the expectation values in Eq (9). While we can use the training data to estimate the first term, we do not have any i.i.d. samples from the unknown model distribution to estimate the second term. Since we are able to compute the conditional probabilities in RBMs efficiently, Gibbs sampling can be used to generate those samples. But Gibbs sampling only guarantees to generate samples from the model distribution if we run it infinitely long. As this is impossible, a finite number of *k* sampling steps are used instead. This procedure is known as the Contrastive Divergence—*k* (CD-*k*) algorithm, in which even *k* = 1 shows good results as shown in [27]. The CD-gradient approximation is given by

$$\frac{\partial \hat{\ell }}{\partial \theta }\approx -{\langle \sum_{h}P\left(h|\tilde{x}\right)\frac{\partial E\left(\tilde{x},h\right)}{\partial \theta }\rangle }_{\tilde{x}}+{\langle \sum_{h}P\left(h|x^{k}\right)\frac{\partial E\left(x^{\left(k\right)},h\right)}{\partial \theta }\rangle }_{x^{\left(k\right)}},$$ (10)

where **x**^(*k*)^ denotes the samples after *k* steps of Gibbs sampling.

The quality of the gradient approximation highly depends on the set of samples used for estimating the model expectation value. Gibbs sampling often has a low mixing rate when used with binary RBMs, which means that the samples tend to stay close to the preceding ones. Therefore, using only a few steps of Gibbs sampling commonly leads to a biased approximation of the gradient [28, 29]. In order to increase the mixing rate Tieleman [30] has suggested to use a persistent Markov chain for drawing samples from the model distribution, which is referred to as persistent Contrastive Divergence (PCD). Desjardins et al. [23] have proposed to use parallel tempering (PT), a method that selects samples from a persistent Markov chain with a different scaling of the energy function. While the advantages of those sampling methods have been shown for binary RBMs, it is not clear if they also transfer to GRBMs.

After computing the derivatives of the energy function with respect to the parameters (see Wang et al. [24]), the corresponding gradient approximations Eq (10) become

$$\frac{\partial \hat{\ell }}{\partial b}\approx {\langle \frac{\tilde{x}-b}{\sigma^{2}}\rangle }_{\tilde{x}}-{\langle \frac{x^{\left(k\right)}-b}{\sigma^{2}}\rangle }_{x^{\left(k\right)}},$$ (11)

$$\frac{\partial \hat{\ell }}{\partial c}\approx {\langle P\left(h=1|\tilde{x}\right)\rangle }_{\tilde{x}}-{\langle P\left(h=1|x^{\left(k\right)}\right)\rangle }_{x^{\left(k\right)}},$$ (12)

$$\frac{\partial \hat{\ell }}{\partial w}\approx {\langle \frac{\tilde{x}P{\left(h=1|\tilde{x}\right)}^{T}}{\sigma^{2}}\rangle }_{\tilde{x}}-{\langle \frac{x^{\left(k\right)}P{\left(h=1|x^{\left(k\right)}\right)}^{T}}{\sigma^{2}}\rangle }_{x^{\left(k\right)}},$$ (13)

$$\begin{array}{ccc} \frac{\partial \hat{\ell }}{\partial \;\sigma } & \approx & {\langle \frac{∥\tilde{x}-b{∥}^{2}-2\;{\tilde{x}}^{T}W\;P\left(h=1|\tilde{x}\right)}{\sigma^{3}}\rangle }_{\tilde{x}}\\ & & -{\langle \frac{∥x^{\left(k\right)}-b{∥}^{2}-2\;{x^{\left(k\right)}}^{T}W\;P\left(h=1|x^{\left(k\right)}\right)}{\sigma^{3}}\rangle }_{x^{\left(k\right)}}, \end{array}$$ (14)

where *P*(**h** = 1|**x**) ≔ (*P*(*h*_1_ = 1|**x**), ⋯, *P*(*h*_*N*_ = 1|**x**))^*T*^, i.e. *P*(**h** = 1|**x**) denotes a vector of probabilities.

The complexity of a single step of MLE for RBM training is O(KDMN), where *K* is the number of Gibbs sampling steps, *D* is the number of data points (batch-size), *M* is the number of visible units, and *N* is the number of hidden units. This can been seen as follows: In each iteration the parameters are updated according to Eqs (11–14) and *K* steps of block Gibbs sampling are performed. The complexity of the parameter update is dominated by the complexity of Eqs (13) and (14), which is O(DMN). For *K* steps of block Gibbs sampling the conditional probabilities of the visible units given the hidden units Eq (6) are evaluated *K* times and the conditional probabilities of the visible units given the hidden units Eq (6) are evaluated *K* + 1 times, which both have a complexity of O(DMN). Therefore, the overall complexity of one step of MLE for training RBMs using *K*-step block Gibbs sampling is given by O(2(K+1)DMN) ~ O(KDMN).

### The marginal probability distribution of the visible units

From the perspective of density estimation, the performance of the model can be assessed by examining how well the model estimates the data distribution. We therefore take a look at the model’s marginal probability distribution of the visible units, which can be formalized as a product of experts (PoE) or as a constrained mixture of Gaussians (MoG).

#### In the form of a product of experts

We derive the marginal probability distribution of the visible units *P*(**X**) by factorizing the joint probability distribution over the hidden units.

$$P\left(X\right)=\sum_{h}P\left(X,h\right)$$ (15)

$$\overset{\left(1,4\right)}{=}\frac{1}{Z}\;e^{-\frac{∥X-{b∥}^{2}}{2\sigma^{2}}}\prod_{j}^{N}\sum_{h_{j}}e^{c_{j}h_{j}+\frac{X^{T}w_{*j}}{\sigma^{2}}h_{j}}$$ (16)

$$\overset{h_{j}\in \left\{0,1\right\}}{=}\frac{1}{Z}\prod_{j}^{N}\left(\;e^{-\frac{∥X-{b∥}^{2}}{2N\sigma^{2}}}+e^{c_{j}+\frac{X^{T}w_{*j}}{\sigma^{2}}-\frac{∥X-{b∥}^{2}}{2N\sigma^{2}}}\right)$$ (17)

$$\begin{array}{ccc} & \overset{\left(21\right)}{=} & \frac{1}{Z}\prod_{j}^{N}\left(\;e^{-\frac{∥X-{b∥}^{2}}{2N\sigma^{2}}}+e^{\frac{∥b+Nw_{*j}{∥}^{2}-{∥b∥}^{2}}{2N\sigma^{2}}+c_{j}-\frac{∥X-b-Nw_{*j}{∥}^{2}}{2N\sigma^{2}}}\right) \end{array}$$ (18)

$$\begin{array}{l} =\frac{1}{Z}\prod_{j}^{N}{\left(\sqrt{2\pi N\sigma^{2}}\right)}^{M}[N(X;b,N\sigma^{2})\\ \;+e^{\frac{||b+Nw_{*j}|{|}^{2}-||b|{|}^{2}}{2N\sigma^{2}}+c_{j}}N(X;b+Nw_{*j},N\sigma^{2})] \end{array}$$ (19)

$$≕\;\frac{1}{Z}\prod_{j}^{N}p_{j}\left(X\right).$$ (20)

From Eqs (17) to (18) we used the relation

$$\begin{array}{ccc} \frac{ax}{\sigma^{2}}-\frac{{\left(x-b\right)}^{2}}{2\sigma^{2}} & = & \frac{-x^{2}+2bx+2ax-b^{2}}{2\sigma^{2}}\\ & = & \frac{-x^{2}+2bx+2ax-b^{2}+a^{2}-a^{2}+2ab-2ab}{2\sigma^{2}}\\ & = & \frac{-{\left(x-a-b\right)}^{2}+a^{2}+2ab}{2\sigma^{2}}. \end{array}$$ (21)

**Fig 1:**
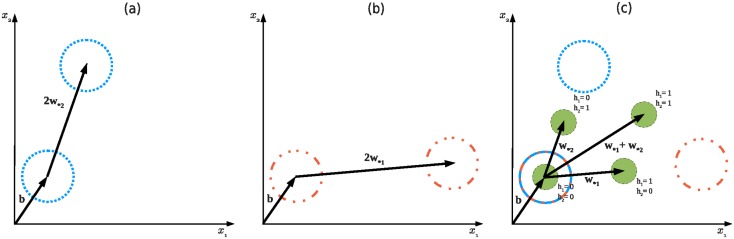
Illustration of a GRBM-2-2 from the perspective of a PoE and a MoG. Arrows indicate the roles of the visible bias vector and the weight vectors. (a) and (b) visualize the two experts of the GRBM. The red (dotted) and blue (dashed) circles indicate the two Gaussians each expert has. (c) visualizes the components in the GRBM seen as a MoG. The components are denoted by the green (filled) circles, and result from the product of the two experts. Notice how each component sits right between a red (dotted) and a blue (dash-dotted) circle.

Eq (20) illustrates that *P*(**X**) can be written as a product of *N* factors, referred to as a product of experts [27]. Each expert *p*_*j*_(**X**) consists of two isotropic Gaussians with the same variance *Nσ*^2^. The first Gaussian is placed at the visible bias **b**. The second Gaussian is shifted relative to the first one by *N* times the weight vector w_*j_ and scaled by a factor that depends on w_*j_ and **b**. Every hidden unit leads to one expert, each mode of which corresponds to one state of the corresponding hidden unit. Fig 1(a) and 1(b) illustrate *P*(**X**) of a GRBM-2-2 viewed as a PoE, where GRBM-*M*-*N* denotes a GRBM with *M* visible and *N* hidden units.

#### In the form of a mixture of Gaussians

Using Bayes’ theorem, the marginal probability of **X** can also be formalized as:

$$P\left(X\right)=\sum_{h}P\left(X|h\right)P\left(h\right)$$ (22)

$$\overset{\left(6,30\right)}{=}\sum_{h}N\left(X;b+Wh,\sigma^{2}\right)\frac{{\left(\sqrt{2\pi \sigma^{2}}\right)}^{M}}{Z}\;e^{c^{T}h+\frac{∥b+{Wh∥}^{2}-{∥b∥}^{2}}{2\sigma^{2}}}$$ (23)

$$\begin{array}{ccc} & \overset{h_{j}\in \left\{0,1\right\}}{=} & \underset{P(h^{\left(\right)})}{\underset{︸}{\frac{{\left(\sqrt{2\pi \sigma^{2}}\right)}^{M}}{Z}}}\;N\left(X;b,\sigma^{2}\right)\\ & & +\sum_{j=1}^{N}\underset{P(h^{\left(j\right)})}{\underset{︸}{\frac{{\left(\sqrt{2\pi \sigma^{2}}\right)}^{M}}{Z}\;e^{\frac{∥b+w_{*j}{∥}^{2}-{∥b∥}^{2}}{2\sigma^{2}}+c_{j}}}}N\left(X;b+w_{*j},\sigma^{2}\right)\\ & & +\sum_{j=1}^{N-1}\sum_{k>j}^{N}\underset{P(h^{\left(j,k\right)})}{\underset{︸}{\frac{{\left(\sqrt{2\pi \sigma^{2}}\right)}^{M}}{Z}\;e^{\frac{∥b+w_{*j}+w_{*k}{∥}^{2}-{∥b∥}^{2}}{2\sigma^{2}}+c_{j}+c_{k}}}}\\ & & N\left(X;b+w_{*j}+w_{*k},\sigma^{2}\right)+\ldots , \end{array}$$ (24)

where **h**^(*j*_1_, *j*_2_, …, *j*_*N*_)^ denotes the binary vector having zero entries except for the dimensions *j*_1_, *j*_2_, …, *j*_*N*_, which are set to one.

*P*(**H**) in Eq (23) is derived as follows

$$P\left(H\right)=\int P\left(x,H\right)dx$$ (25)

$$\overset{\left(1,4\right)}{=}\frac{1}{Z}\int e^{c^{T}H}\prod_{i}^{M}e^{\frac{x_{i}w_{i*}^{T}H}{\sigma^{2}}-\frac{∥x_{i}-b_{i}{∥}^{2}}{2\sigma^{2}}}dx$$ (26)

$$=\frac{e^{c^{T}H}}{Z}\prod_{i}^{M}\int e^{\frac{x_{i}w_{i*}^{T}H}{\sigma^{2}}-\frac{∥x_{i}-b_{i}{∥}^{2}}{2\sigma^{2}}}dx_{i}$$ (27)

$$\overset{\left(21\right)}{=}\frac{e^{c^{T}H}}{Z}\prod_{i}^{M}\left(e^{\frac{{\left(b_{i}+w_{i*}^{T}H\right)}^{2}-b_{i}^{2}}{2\sigma^{2}}}\int e^{\frac{∥x_{i}-b_{i}-w_{i*}^{T}H{∥}^{2}}{2\sigma^{2}}}dx_{i}\right)$$ (28)

$$=\frac{e^{c^{T}H}}{Z}{\left(\sqrt{2\pi \sigma^{2}}\right)}^{M}e^{\sum_{i}^{M}\frac{{\left(b_{i}+w_{i*}^{T}H\right)}^{2}-b_{i}^{2}}{2\sigma^{2}}}$$ (29)

$$=\frac{{\left(\sqrt{2\pi \sigma^{2}}\right)}^{M}}{Z}\;e^{c^{T}H+\frac{∥b+{WH∥}^{2}-{∥b∥}^{2}}{2\sigma^{2}}}$$ (30)

Since the form of Eq (24) is identical to a mixture of isotropic Gaussians, we follow its naming convention. Each Gaussian distribution is called a *component* of the model distribution, which is exactly the conditional probability of the visible units given a particular state of the hidden units. Like in MoGs, each component has a *mixing coefficient*, which is the marginal probability of the corresponding hidden state and can also be viewed as the prior probability of picking the corresponding component. The total number of components in a GRBM is 2^*N*^, which is exponential in the number of hidden units, see Fig 1(c) for an example.

The locations of the components in a GRBM are not independent of each other as it is the case in MoGs. They are centered at **b** + **Wh**, which is the vector sum of the visible bias and selected weight vectors. The selection is done by the corresponding entries in **h** taking the value one. This implies that only the *M* + 1 components that sum over exactly one or zero weights can be placed and scaled independently. We name them first order components and the anchor component, respectively. All 2^*N*^ − *M* − 1 higher order components are then determined by the choice of the anchor and first order components, showing that GRBMs are constrained MoGs with isotropic components.

## Experiments

### Two-dimensional blind source separation

The general assumption in the analysis of natural images is that they can be considered as a mixture of independent super-Gaussian sources [31], (but see [32] for an analysis of remaining dependencies). We therefore use a mixture of two independent Laplacian distributions as a toy example, to visualize how GRBMs model natural image statistics.

The independent sources **s** = (*s*_1_, *s*_2_)^*T*^ with $p(s_{i})=\frac{e^{-\sqrt{2}\left|s_{i}\right|}}{\sqrt{2}}$ are mixed by a random mixing matrix **A** yielding

$${\tilde{x}}^{\prime }=As.$$ (31)

It is common to whiten the data, resulting in

$$\tilde{x}=V{\tilde{x}}^{\prime }=VAs,$$ (32)

where $V={\langle \tilde{x^{\prime }}{\tilde{x^{\prime }}}^{T}\rangle }^{-\frac{1}{2}}$ is the whitening matrix calculated with principle component analysis (PCA). Throughout this paper, we used whitened data.

In order to assess the performance of GRBMs in modeling the data distribution, we trained GRBMs with two visible and two hidden units (GRBM-2-2) and GRBMs with two visible and four hidden units (GRBM-2-4) on the toy problem using CD-1 with a learning rate of 0.1. For comparison, we also fitted two-dimensional isotropic Gaussian distributions to the data distribution and trained ICA models using the FastICA algorithm [33]. FastICA has a complexity of O(2DM(M+1))~O(DMM) [34], where D is the number of data points (batch-size), and M is the data dimensionality. Thus, the asymptotic complexity of FastICA and MLE for RBM training is comparable, and becomes equivalent if the number of visible and hidden units are the same (*M* = *N*) and the number of Gibbs sampling steps is set to one (*K* = 1).

All experiments were repeated 200 times and we calculated the average $\hat{\ell }$ over the test data for all models. For the super-Gaussian sources of ICA, $\hat{\ell }$ can be assessed analytically by

$$\hat{\ell }=-{\langle \sum_{j=1}^{N}\ln\tilde{p}(w_{*j}^{T}{\tilde{x}}_{l})\rangle }_{{\tilde{x}}_{l}}+\ln\left|\det W\right|.$$ (33)

$$=-{\langle \sum_{j=1}^{N}\ln2\cosh^{2}\;w_{*j}^{T}{\tilde{x}}_{l}\rangle }_{{\tilde{x}}_{l}}+\ln\left|\det W\right|,$$ (34)

where $\tilde{p}\left(s_{i}\right)=2\cosh^{2}\left(s_{i}\right)$ is used by the fast ICA algorithm as a smooth approximation of *p*(*s*_*i*_). Furthermore, as we know the true data distribution, the exact $\hat{\ell }$ can also be calculated by

$$\hat{\ell }=-\sqrt{2}{\langle |u_{1*}{\tilde{x}}_{l}\left|+\right|u_{2*}{\tilde{x}}_{l}|\rangle }_{{\tilde{x}}_{l}}-\ln2+\ln\left|\det U\right|,$$ (35)

where **U** = (**VA**)^−1^. The results are presented in Table 1, which confirm the conclusion of [11] that GRBMs are not as good as ICA in terms of $\hat{\ell }$ if the same number of visible and hidden units are used. For a GRBM-2-4, however, $\hat{\ell }$ comes close to that of of ICA.

**Table 1:** Comparison of $\hat{\ell }$ for different models trained on the blind source separation task.

Method	$\hat{\ell }\pm \text{std}$
Gaussian	−2.8367 ± 0.0086
GRBM-2-2	−2.8072 ± 0.0088
GRBM-2-4	−2.7448 ± 0.0125
ICA	−2.7382 ± 0.0091
Data distribution	−2.6923 ± 0.0092

**Fig 2:**
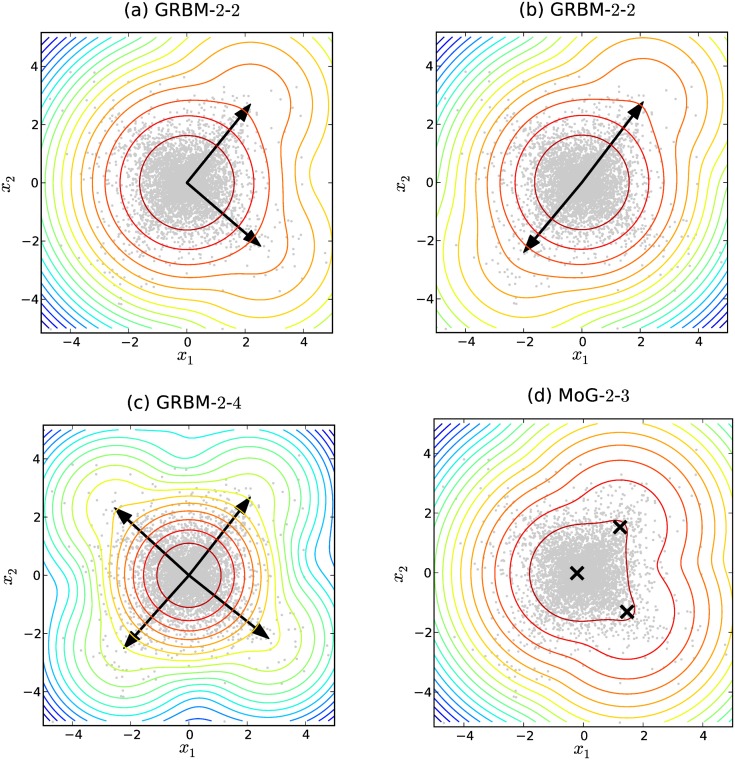
Illustration of the log-probability densities for models trained on the blind source separation task. The data is plotted as gray dots. (a) shows a GRBM-2-2 that has learned two independent components. (b) shows a GRBM-2-2 that has learned one independent component with opposite directions. (c) shows a GRBM-2-4. (d) shows an isotropic MoG with three components. The arrows indicate the weight vectors of the GRBMs, while the crosses denote the means of the MoG components. Comparing (a) and (d), the contribution of the second order component is so insignificant that the probability distribution of the GRBM with four components is almost the same as the MoG with three components.

**Fig 3:**
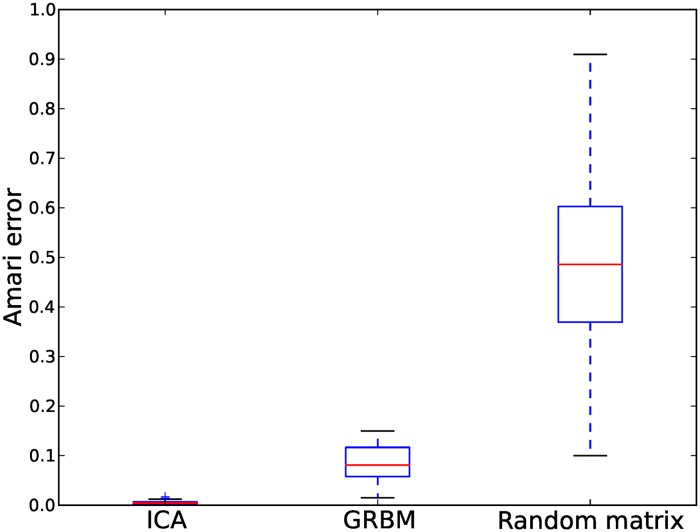
Amari errors of ICA and GRBM-2-2 on the blind source separation task over different trials. The Amari error compares the estimated with the true unmixing matrix. The box extends from the lower to the upper quantile values of the errors, with a line at the median. The whiskers extending from the box show the minimum-maximum range. As a base line, the Amari errors between the real unmixing matrices and random matrices are provided.

To illustrate how GRBMs model the statistical structure of the data, we looked at the probability distributions and the weight vectors of the 200 trained GRBMs. About half of them (110 out of 200) recovered the independent components, see Fig 2(a) as an example. This can further be illustrated by plotting the Amari error between the true unmixing matrix **A**^−1^ and estimated model matrices, i.e. the unmixing matrix of ICA and the weight-matrix of the GRBM-2-2, as shown in Fig 3. The Amari error [35] between two matrices **A** and **B** is defined as

$$\frac{1}{2N}\left(\sum_{i=1}^{N}\sum_{j=1}^{N}\frac{|{\left(AB^{-1}\right)}_{ij}|}{\max_{k}|{\left(AB^{-1}\right)}_{ik}|}+\frac{|{\left(AB^{-1}\right)}_{ij}|}{\max_{k}|{\left(AB^{-1}\right)}_{kj}|}\right)-1.$$ (36)

One can see that these 110 GRBMs estimated the unmixing matrix quite well, although GRBMs are not as good as ICA. This is due to the fact that the weight vectors in GRBMs are not restricted to be orthogonal as in ICA.

For the remaining 90 GRBMs, the two weight vectors pointed to the opposite direction as shown in Fig 2(b). Accordingly, these GRBMs failed to estimate the unmixing matrix, but in terms of density estimation these solutions have the same quality as the orthogonal ones. Thus all the 200 GRBMs were able to learn the statistical structure in the data and model the data distribution pretty well.

For comparison, we plotted the probability distribution of a learned GRBM with four hidden units, see Fig 2(c), in which case the GRBMs always find the two independent components correctly.

To further show how the components contribute to the model distribution, we randomly chose one of the trained GRBM-2-4, GRBM-2-4, and MoG-2-3 model and calculated the mixing coefficients of the anchor and the first order components, as shown in Table 2. The large mixing coefficient for the anchor component indicates that the model most likely reaches hidden states in which none of the hidden units is activated. In general, the more activated hidden units a state has the less likely it is reached, which leads naturally to a sparse representation of the data.

**Table 2:** Sums of the mixing coefficients for models trained on the blind source separation task.

Method	$\sum_{h\in H_{0}}P\left(h\right)$	$\sum_{h\in H_{1}}P\left(h\right)$	$\sum_{h\in H_{2}}P\left(h\right)$	$\sum_{h\in H_{3}}P\left(h\right)$	$\sum_{h\in H_{4}}P\left(h\right)$
GRBM-2-2	0.9811	0.0188	7.8856*e*-05	–	–
GRBM-2-4	0.9645	0.0352	3.4366*e*-04	1.2403*e*-10	6.9977*e*-18
MoG-3	0.9785	0.0215	–	–	–

Sums of the mixing coefficients of a successfully trained GRBM-2-2, GRBM-2-4 and a MoG-3. $H_{0}$ denotes the set containing only the vector with zero entries, $H_{1}$ denotes the set of all vectors where exactly one entry is set to one and the rest is set to zero, $H_{2}$ denotes the set of all vectors where exactly two entries are set to one and the rest is set to zero and so forth.

The dominance of the anchor mixing coefficient ($\sum_{h\in H_{0}}P\left(h\right)$) and the first order mixing coefficients ($\sum_{h\in H_{1}}P\left(h\right)$) as shown in Table 2, can also be seen in Fig 2 by comparing a GRBM-2-2 (a) with a two dimensional MoG having three isotropic components denoted by MoG-2-3 (d). Although the MoG-2-3 has one component fewer than the GRBM-2-2, the two models have almost the same probability distribution. The first order components of the GRBM-2-2, however, have a greater distance to the anchor component than those of the MoG-2-3, which is necessary to achieve the very small mixing coefficients of the second order component.

### Natural image patches

In contrast to random images, natural images have a common underlying structure which could be used to code them more efficiently than with a pixel-wise representation. Olshausen and Field [36] have shown that sparse coding is such an efficient coding scheme and that it is in addition a biologically plausible model for the simple cells in the primary visual cortex. Bell and Sejnowski [31] have shown that the independent components provide a comparable representation for natural images. We now want to test empirically whether GRBMs, like sparse coding and ICA, generate such biologically plausible results.

We used the Van Hateren’s natural image database [37] and randomly sampled 70,000 grey scale image patches with a size of 14 × 14 pixels. The mean of each image patch was removed separately and then whitened using Zero-phase Component Analysis (ZCA). Afterwards the data set was divided into 40,000 training and 30,000 testing image patches. Training a GRBM on natural image patches is not a trivial task and we followed the recipes discussed in detail in the next section.

**Fig 4:**
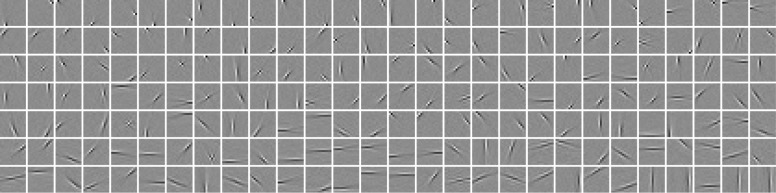
Illustration of 196 learned filters learned by a GRBM-196-196. The plot has been ordered from left to right and from top to bottom by the increasing average activation level of the corresponding hidden units.

In Fig 4, we display the learned weight vectors w_*j_, named features or filters, which can be regarded as receptive fields of the hidden units. They are fairly similar to the filters learned by ICA [31].

**Fig 5:**
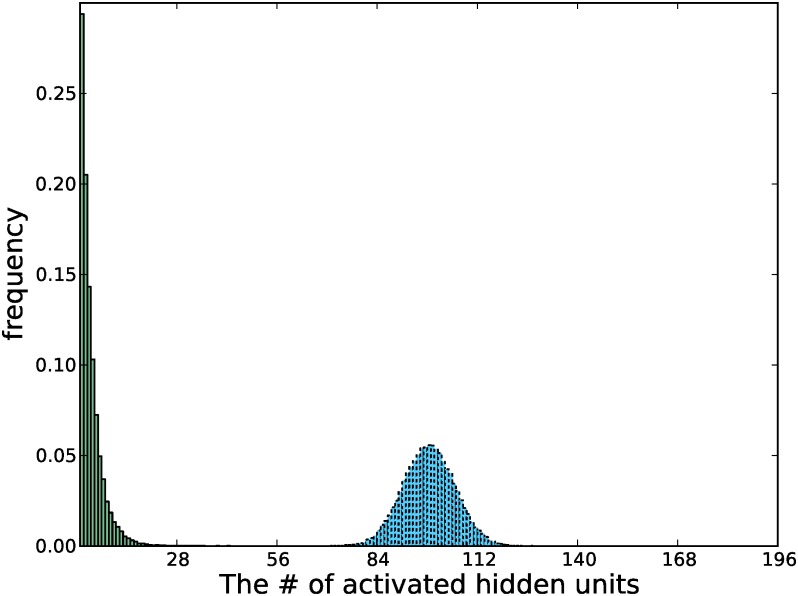
Histogram of the number of activated hidden units of a GRBM-196-196. The model was trained on whitened natural image patches. The histograms before and after training are plotted in blue (dotted) and in green (solid), respectively.

Like in the 2D experiments, we calculated the anchor and first order mixing coefficients, as shown in Table 3. The coefficients are much smaller compared to the anchor and first order coefficients of the GRBMs in the two dimensional case. However, they are still significantly large, considering that the total number of components in this case is 2^196^. Like in the 2D experiments, the more activated hidden units a state has, the less likely it will be reached, which leads naturally to a sparse representation. To support this statement, we plotted the histogram of the number of activated hidden units per training sample, as shown in Fig 5.

**Table 3:** Sums of the mixing coefficients of a GRBMs-196-196 trained on whitened natural image patches.

Method	$\sum_{h\in H_{0}}P\left(h\right)$	$\sum_{h\in H_{1}}P\left(h\right)$	$\sum_{h\in H\backslash \{H_{0}\cup H_{1}\}}P\left(h\right)$
GRBM-196-196	0.04565	0.00070	0.95365

$H_{0}$ denotes the set containing only the vector with zero entries, $H_{1}$ denotes the set of all vectors where exactly one entry is set to one and the rest is set to zero, and $H\backslash \{H_{0}\cup H_{1}\}$ represents the set of all remaining vectors. (the Partition function was estimated using annealed importance sampling).

**Fig 6:**
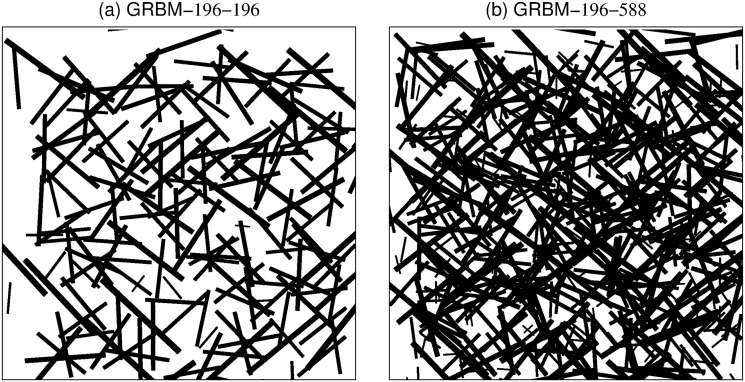
Spatial layout and size of the filters learned on whitened natural image patches. (a) for a GRBM-196-196 and (b) for a GRBM-196-588. The layout and size of the filters represented by the position and size of the bars. Each bar denotes the center position and the orientation of a Gabor function fitted to one of the learned filters. Thickness and length of each bar are proportional to the spatial-frequency bandwidth of the corresponding filters.

**Fig 7:**
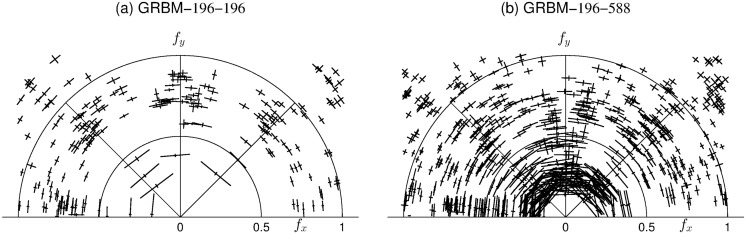
Polar plot of frequency tuning and orientation of the filters learned on whitened natural image patches. (a) for a GRBM-196-196 and (b) for a GRBM-196-588. The crosshairs describe the selectivity of the filters, which is given by the 1/16-bandwidth in spatial-frequency and orientation, see [38] for details.

We also examined the results of GRBMs in the over-complete case, i.e. GRBM-196-588. There is no prominent difference of the filters compared to the complete case shown in Fig 4. To further compare the filters in the complete and over-complete case, we estimated and plotted the spatial frequency, location and orientation for all filters in the spatial and frequency domain, see Figs 6 and 7, respectively. This is achieved by fitting a Gabor function of the form used by Lewicki and Olshausen [38]. In the over-complete case the space of locations and frequencies is covered more densely.

For comparison, we also trained ICA on the whitened natural image data set, which achieved an $\hat{\ell }$ averaged over 10 trials of -259.19 on the training data and -259.66 on the test data (see Table 4). Like in the 2D experiments, the average $\hat{\ell }$ of GRBMs became comparable to that of ICA if we doubled the number of hidden units. A GRBM-196-392 achieved an $\hat{\ell }$ averaged over 10 trials of -257.78 on the training data and -260.40 on the test data after 1000 epochs of training, which only changed slightly to -257.38 on the training data and -260.03 on the test data after 2000 epochs of training as shown in Table 4. We also trained a MoG with nine Gaussian components each having a free covariance matrices, which achieved an $\hat{\ell }$ of -229.35 on the training data and -241.87 on the test data (see Table 4). Our results are consistent with the findings of Theis et al. [11], that a MoG with only a few components and free covariance matrices lead to a better $\hat{\ell }$ than ICA and GRBMs. However, the resulting filters i.e. the eigenvectors of the covariance matrix are not localized edge detectors such as in ICA or GRBMs, see Melchior [18] for a visual comparison.

**Table 4:** Comparison of $\hat{\ell }$ on training and test data for different models trained on whitened natural image patches.

Method	$\hat{\ell }$ train	$\hat{\ell }$ test
ICA	−259.19	−259.66
GRBM-196-392	−257.38	−260.03
Mixture of 9 Gaussians (MoG-9)	−229.35	−241.87

## Successful training of GRBMs on natural images

Training GRBMs has been reported to be difficult [8, 9]. Based on our analysis we propose several modifications on the training setup that in our experiments improve the success and speed of training GRBMs on natural image patches significantly. Some of them do not depend on the data distribution and should therefore improve training in general.

### Preprocessing of the data

Preprocessing the data is important especially if the model is highly restricted like GRBMs. Whitening is a common preprocessing step for natural images. It removes the first and second order statistics from the data so that it has zero mean and unit variance in all directions. This allows training algorithms to focus on higher order statistics like kurtosis, which is presumed to play an important role in natural image representations [36, 39].

The components of GRBMs are isotropic Gaussians, such that the model would need several components to model covariances. But the whitened data has a spherical covariance matrix, such that the distribution can be modeled already fairly well by a single component. The other components can then be used to model higher order statistics, so that we claim that whitening is an important preprocessing step for GRBMs.

### Parameter initialization

The initial choice of the model parameters is important for the optimization process. Using prior knowledge about the optimization problem can help to derive an initialization that can improve speed and success of the training.

For GRBMs we know from the analysis above that the anchor component, which represents most of the whitened data moves to the data mean during training. Therefore, it is reasonable in practice to set the visible bias to the value of the data mean without training it.

Learning the right scaling is usually very slow since weights and biases jointly determine the position and scaling of the components. In the final stage of training GRBMs on whitened natural images, the first order components are scaled down extremely compared to the anchor component, since the data distribution is rather dense in the region of the first order components. Therefore, it will usually speed up the training process if we initialize the parameters so that the first order scaling factors are already very small. Considering Eq (24), we are able to set a specific first order scaling factor by initializing the hidden bias to

$$c_{j}=-\frac{∥b+w_{*j}{∥}^{2}-{∥b∥}^{2}}{2\sigma^{2}}+\ln\tau_{j},$$ (37)

so that the scaling is determined by *τ*_*j*_, which should ideally be chosen close to the unknown final scaling factors. In practice, the choice of 0.01 showed good performance in most cases.

Furthermore, the initial norms of the weight-matrix columns should also be comparable to the corresponding norms of a successfully trained GRBM. A common way however is to initialize the weights with small Gaussian-distributed random values, in which case the norms of the weight-matrix columns are small, such that all components are positioned close to the anchor component. According to Bengio and Glorot [40], the weights of a artificial neural network should be initialized to $w_{ij}∼U(-\frac{\sqrt{6}}{\sqrt{N+M}},\frac{\sqrt{6}}{\sqrt{N+M}})$, where *U*(*a*, *b*) is the uniform distribution in the interval [a, b]. This initialization leads to much larger norms of the weight-matrix columns, which in our experience works better than the commonly used Gaussian-distributed random values. In combination with the proposed initialization for the biases, the component scaling and the norms of the weight-matrix columns are close to the values of a successfully trained GRBM, so that the optimization problem becomes more about finding the right rotation of the weight vectors rather than their position and scaling.

### Gradient restriction and choice of hyperparameter

The choice of the hyperparameters has a significant impact on the speed and success of training GRBMs. For successful training in an acceptable number of parameter updates, the learning rate needs to be sufficiently large otherwise the learning process becomes too slow or the algorithm converges to a local optimum where all components are placed in the data mean. But if the learning rate is chosen too high the norm of the parameter updates often get too large leading to a divergence of $\hat{\ell }$. This effect becomes even more critical as the model dimensionality increases. In our experience, for a GRBM with 196 visible and 1,000 hidden units, $\hat{\ell }$ diverges already for a learning rate of 0.001. This problem usually leads to the choice of a rather small learning rate, which in turn leads to a rather slow learning speed.

To prevent divergence, we propose to restrict the norm of the weight gradient columns ∇*w*_:*j*_ to a reasonable size. Since we know from our analysis that the components are placed in the region of data and that the position of the first order components are determined by the corresponding weight-matrix column, there is no need for a norm of the weight-matrix columns lager than twice the maximal data norm. This bound also holds for the gradient and should be chosen even smaller to prevent the components from changing their position to quickly. In practice, one should restrict the column norms of the update matrix rather than column norms of the gradient matrix to also account for the effects of a momentum term for example. The restriction allows us to use large learning rates even for very large models and therefore enables fast and robust training. In our experience a value of one hundredth of the maximal data norm yielded good performance in general.

The batch-size should be chosen large enough, so that the entire data set is sufficiently represented by the data points within each batch. In practice a batch-size of 100 or larger yielded good results.

A momentum term adds a percentage of the old gradient to the current gradient, which can lead to faster and more robust learning especially for small batch-sizes. We did not observe a benefit of using a momentum term compared to simply using a larger learning rate if the batch-size is chosen rather large (i.e. ≥100).

Since the components are placed on the data they are naturally restricted. This makes the use of a weight decay useless or even counter productive since it prevents the system from converging to the optimal solution where the weight columns have a certain norm.

The use of a sparse penalty is not necessary since we know from our analysis that a sparse representation emerges naturally when GRBMs are trained on whitened natural image patches. Furthermore, when using a sparse penalty we have to guess the unknown sparseness level in advance, which might differ significantly from the optimal value.

### Results

**Fig 8:**
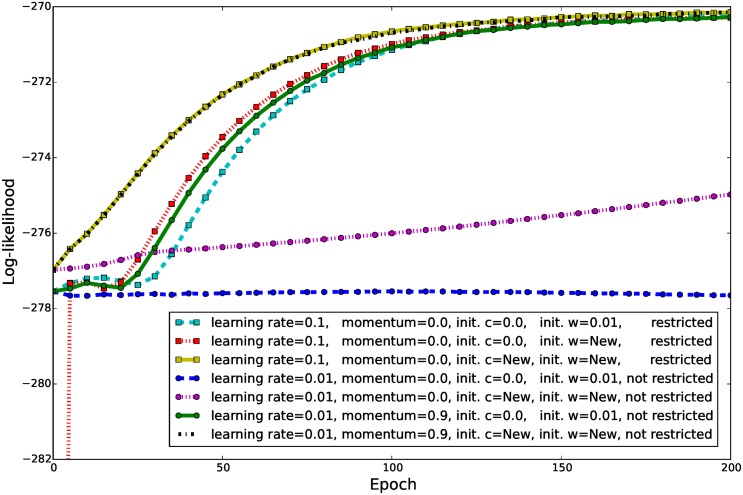
Evolution of $\hat{\ell }$ on natural image patches when using different training setups. GRBM-196-196s were trained on whitened natural image data set with CD-1. The learning curves are the average $\hat{\ell }$ on the test data over 10 trials and $\hat{\ell }$ was estimated using AIS. “init. c = New” and “init. w = New” correspond to the initializations proposed in the previous section, and “init. w = 0.01” corresponds to an initialization of the weights to Gaussian distributed random values with standard deviation of 0.01. The learning rate for the variance parameter was set 100 times smaller than for the other parameters.

To show the effect of the training setups proposed, we trained several GRBMs with 196 hidden units on the whitened natural image data set for 200 epochs using CD-1 with different parameter initializations and training setups. Since the model is very sensitive to changes in the variance parameter, we set the corresponding learning rate to be 100 times smaller than the learning rate for the weights and bias in all experiments. Furthermore, we set the visible bias to the data mean without updating it. Each experiment was repeated 10 times and in each trial $\hat{\ell }$ was estimated every fifth epoch using 100 repetitions of annealed importance sampling [41](AIS) with 10,000 linearly distributed inverse temperatures. The evolution of the average $\hat{\ell }$ on the test data, for the different experiments described in the following is shown in Fig 8. In all experiments, the evolution of $\hat{\ell }$ on the training data (not shown) and test data were qualitatively the same.

In a first experiment, we used a training setup as it is often used in practice, i.e. a learning rate of 0.01, a visible and hidden bias initialized to zero, and a weight-matrix initialized to small Gaussian distributed random values. The model did not capture the statistics of the data since the visualized filters (not shown) look just like noise patterns and $\hat{\ell }$ did not increase over 200 epochs (see Fig 8). Increasing the learning rate to 0.1 led instantaneously to an extreme divergence of $\hat{\ell }$ independently of the chosen initialization and whether a momentum was used or not. We therefore performed the same experiment with a learning rate of 0.1 but restricted the parameter updates to one hundredth of the maximal data norm, which led to a significant improvement of $\hat{\ell }$ (see Fig 8). The learned filters looked similar to those shown in Fig 4. We also performed experiments where we changed the initialization of the weight-matrix and the hidden bias to the initialization proposed in the previous section. In both cases the quality of the learned filters and the final $\hat{\ell }$ stayed almost the same. The learning speed, however, increased when the weights are initialized as proposed by Bengio and Glorot [40] (indicated by “init w = New” in Fig 8) and increased even more when in addition our proposed initialization for the hidden bias values is used (indicated by “init c = New” in Fig 8). As control experiments we also trained models using the proposed initialization with a smaller learning rate of 0.01 but without gradient restriction. As shown in Fig 8, $\hat{\ell }$ did not diverge but the speed of convergence became rather slow and the model did not get even close to convergence after 200 epochs. When in addition a momentum term of 0.9 was used, the performance became almost equivalent to the performance when the model is trained with a learning rate of 0.1 and gradient restriction. However, when the learning rate was further increased or the number of hidden units was increased the divergence of $\hat{\ell }$ was observed again.

The advantage of PCD and PT sampling for training binary RBMs has been shown in several studies [23, 30, 42]. For GRBMs, Cho et al. [9] have shown some advantage of PT over CD sampling in terms of the reconstruction error and classification rate. But to our knowledge there exists no analysis of the different sampling algorithms with respect to the objective being optimized, which is $\hat{\ell }$. We therefore performed a second set of experiments where we compared different sampling algorithms for training GRBMs on whitened natural image patches. We used the gradient restriction, our proposed initialization for the hidden bias and the weight initialization proposed by Bengio and Glorot [40]. All models were trained for 400 epochs and $\hat{\ell }$ was estimated every 10th epoch using AIS with the same setup as described above. We use (P)CD-1 and (P)CD-10 to denote the use of (P)CD sampling with either 1 or 10 sampling steps. For PT we used k = 1 with 10 linearly distributed inverse temperatures from 0 to 1 denoted by PT-1_10_.

**Fig 9:**
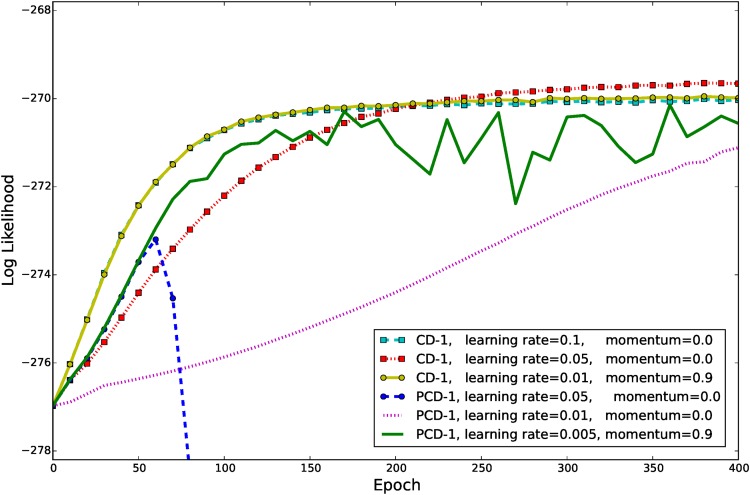
Evolution of $\hat{\ell }$ on whitened natural image patches when using different training methods. GRBM-196-196s were trained on the whitened natural image data set using CD-1 and PCD-1 using different learning rates and momentum terms. The learning curves are the average over 10 trials. The gradient was restricted to one hundredth of the maximal data norm (0.48) and the learning rate for the variance parameter was set 100 times smaller than for the other parameters.

Fig 9 shows the evolution of $\hat{\ell }$ when either CD-1 or PCD-1 was used for training in combination with different learning rates. When CD-1 was used with a learning rate of 0.1 the model quickly converged to an average $\hat{\ell }$ around -270. No significant difference can be observed when a learning rate of 0.01 in combination with a momentum term of 0.9 was used instead. A smaller learning rate of 0.05 without momentum led to a more stable convergence and thus to a slightly higher average $\hat{\ell }$, but at the same time reduced the convergence speed.

When using a persistent Markov chain the assumption is that the model distribution changes slowly enough through one parameter update so that the samples after one sampling step are still representative for the updated model distribution. Thus, PCD-1 usually requires a much smaller learning rate than CD-1 [29, 43]. This can also be seen from Fig 9, when PCD-1 was used for training in combination with a learning rate of 0.05, $\hat{\ell }$ immediately diverged. However, when a learning rate of 0.01 was used, $\hat{\ell }$ did not diverge but the speed of convergence reduced significantly. Using an even smaller learning rate in combination with a momentum term did not help to overcome the instability problem as can also be seen in Fig 9 for a learning rate of 0.005 and a momentum term of 0.9.

**Fig 10:**
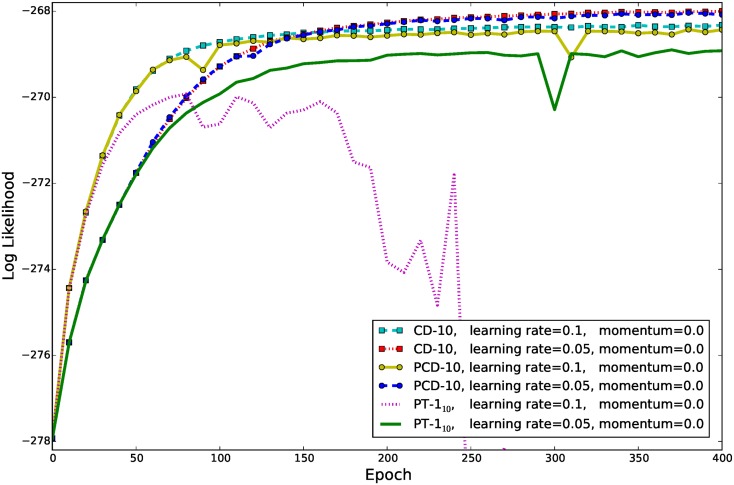
Evolution of $\hat{\ell }$ on whitened natural image patches when using advanced training methods. GRBM-196-196s were trained on the whitened natural image data set using CD-10, PCD-10, and PT-10 with 10 linearly distributed inverse temperatures. The learning curves are averaged over 10 trials. The gradient was restricted to one hundredth of the maximal data norm (0.48) and the learning rate for the variance parameter was set 100 times smaller than for the other parameters.

One way to overcome the divergence problem when using PCD-1, besides using a smaller learning rate, is to use more sampling steps between the parameter updates. Fig 10 shows the evolution of $\hat{\ell }$ for PCD-10, CD-10 and PT-1_10_ using either a learning rate of 0.1 or 0.05. In the case of PCD-10, even a learning rate of 0.1 did not lead to a divergence of $\hat{\ell }$ anymore. For both learning rates the results for CD-10 and PCD-10 were rather similar although the evolution of $\hat{\ell }$ appeared to be more stable in the case of CD-10. Both methods reached a significantly higher $\hat{\ell }$ than CD-1 and PCD-1 and a slightly better $\hat{\ell }$ was reached when a smaller learning rate of 0.05 is used. PT-1_10_ diverged for a learning rate of 0.1 and reached a slightly worse $\hat{\ell }$ than CD-10 and PCD-10 when a learning rate of 0.05 is used. The slightly worse $\hat{\ell }$ of PT might be explained by the fact that, although samples might come form different temperatures, PT still uses one step of sampling between the parameter updates. Compared to CD-1 and PCD-1, however, PT reached a better $\hat{\ell }$ value. We would expect PT-10_10_ also to be better than CD-10 and PCD-10. However, the computational overhead is almost 10 times higher than for CD-10 and PCD-10, which itself have a 10 times higher computational overhead than CD-1 and PCD-1, which makes PT-10_10_ impracticable. Note that initially we trained GRBMs with only 16 hidden units in which case $\hat{\ell }$ did not differ significantly for the different training methods.

Since the computational overhead for the gradient restriction is very small, it is advisable to use it precautionally in general, even though the threshold might never be exceeded during training. Furthermore, it is beneficial to use our proposed initialization of the hidden units as well as the proposed initialization of the weights by Bengio and Glorot [40]. We did not observe any benefit of using a momentum term compared to using a larger learning rate instead. Since PCD and PT do not reach better final $\hat{\ell }$ but are incompatible with large learning rates when a small number of sampling steps is used, it is advisable to use CD instead of PCD. Using a larger *k* is advisable but also increases the computational cost substantially.

## Comparison to related work

Training GRBMs is known to be difficult and several studies [8–19] have addressed this problem using different ways of evaluating the model performance. Most of the studies have investigated the learned filters/features visually, showing comparable features to those shown in Fig 4 or they have compared the performance of classifiers that were trained on the GRBMs feature output. A good discriminative performance, however, does not imply a good generative performance and *vice versa*. Since GRBMs are optimized for the $\hat{\ell }$ objective it is thus rather questionable if classification rates are a good way of evaluating model performances. To our knowledge there is only one publication [11] besides ours that has evaluated and compared the $\hat{\ell }$ for different models including GRBMs. The results of Theis et al. [11] are consistent with our findings that GRBMs with the same number of visible and hidden units have a worse performance than ICA, and that MoGs with only a few Gaussian components and free covariances matrices lead to a better $\hat{\ell }$ than ICA and GRBMs. One should note, however, that the resulting filters of MoGs (i.e. the eigenvectors of the covariance matrix) are not localized edge detectors such as filters learned by ICA or GRBMs, see Melchior [18] for a visual comparison.

We emphasize the importance of training the variance parameter since it changes the performance significantly especially when a large number of hidden units is used [18]. The majority of other studies, however, have used GRBMs with a fixed value for the variance parameter (*i.e.* set to one). Only two studies [8, 9], both using a different parameterization of the energy function, have considered training the variance parameter. While Krizhevsky [8] has concluded that it is rather difficult and thus impractical to train the variance parameter, Cho et al. [9] have also found that training the variance parameter is of importance.

All studies [8–19] except ours have used a rather small learning rate to avoid problems during GRBM training, which leads to a rather slow convergence (as an example see Fig 9). It is thus questionable whether the models have reached convergence and whether reported results change if the models are trained till convergence using our proposed methods. Cho et al. [9] have addressed the training difficulties by proposing a modification of PT sampling and an adaptive learning rate. We claim, however, that the problem of using a large learning rate with PCD or PT sampling can be compensated by restricting the gradient and using a bigger number of sampling steps (see Fig 10). Lee et al. [10] have shown that GRBMs could also learn meaningful filters by using a sparse penalty. We show however that a sparse penalty is not necessary to learn meaningful features and in our experience a penalty usually leads to much worse $\hat{\ell }$ values compared to an unregularized model.

Apart from the analysis of the original model, some extensions of GRBMs have been proposed to overcome reported difficulties. Ranzato et al. [14] have argued that the failure of GRBMs in modeling natural images is due to the model’s focus on predicting the mean intensity of each pixel rather than the dependence between pixels. They have proposed the mean-covariance RBM (mcRBM), which in addition to the conventional hidden units has a group of hidden units that can model the covariance between the visible units. Compared to a GRBM, an mcRBM can have a covariance matrix that is not restricted to be diagonal and can thus be considered as an improved GRBM. Since the visible units of an mcRBM are not conditionally independent, the model cannot be trained by simple block Gibbs sampling anymore. Instead, drawing samples from the model distribution needs to be approximated, which makes training more difficult and costly. The authors have shown that an mcRBM learns filters similar to those of ICA and GRBMs and that mcRBMs lead to features that are more discriminative than those of GRBMs, resulting in a better classification rate on the CIFAR-10 data set. Another explanation for the failure of GRBMs has been provided by Nair et al. [12] as well as Courville et al. [15], who argue that the deficiency of GRBMs in modeling covariances is due to the binary nature of the hidden units. Nair et al. [12] have proposed to replace the binary hidden units by noisy linear rectifier units, which allows the model to learn similar filters to those of GRBMs, but outperforms the original model in terms of classification rates. Courville et al. [15] have developed the spike and slab RBM (ssRBM), which splits each binary hidden unit into a binary spike variable and a real-valued slab variable. According to Fig 1, the real-valued slab variables would allow to shift the components along the corresponding weight vector w_*j_, such that a ssRBM can be considered as a more flexible GRBM. The authors have shown that ssRBMs also learn filters similar to those of ICA and GRBMs and that ssRBMs lead to similar classification rates on CIFAR-10 data set as mcRBMs. A natural extension of an RBM is a deep Boltzmann machine (DBM) [42, 44], which has additional binary hidden layers. In contrast to a deep belief network all layers in a DBM are trained jointly. A Gaussian-binary/Gaussian-Bernoulli DBM (GDBM) [45] has similar properties than the ssRBM and mcRBM in modeling natural image statistics, see Wang [19] for a detailed comparison. We agree on the advantages and higher flexibility of mcRBM, ssRBM, and GDBMs in modeling natural image statistics. One should note, however, that GRBMs might get closer to the results of mcRBMs and ssRBMs if they are trained with a sufficiently large learning rate and if the standard deviation is optimized.

## Conclusion

In this paper, we provide a theoretical analysis of GRBMs and show that its product of experts formulation can be rewritten as a constrained mixture of Gaussians. This representation gives a much better insight into the capabilities and limitations of the model.

We use a two-dimensional blind source separation task as a toy problem to demonstrate how GRBMs model the data distribution. The results illustrate that GRBMs learn meaningful features both for the toy problem and when modeling natural image patches. In both cases, the learned features are comparable to those of ICA, which is generally accepted to be an appropriate model for natural image statistics. Although ICA reaches a better $\hat{\ell }$ than GRBMs that have the same number of visible and hidden units, a comparable $\hat{\ell }$ can be reached by GRBMs with twice as many hidden than visible units. We show on the one hand that a large learning rate is required to train GRBMs in an acceptable number of parameter updates, but on the other hand a large learning rate can easily lead to divergence of $\hat{\ell }$, which we identify as the main reason for reported difficulties in training GRBMs [8–19]. Based on our theoretical analysis, we propose to restrict the norm of the parameter updates to a reasonable size. We illustrate that the restriction of the gradient prevents divergence and allows to use large learning rates for fast and robust training of GRBMs. Furthermore, we propose a better way to initialize the hidden biases leading to an even faster convergence than a naive initialization. Our results suggest that CD learning is more appropriate when training GRBMs on natural images than sampling methods that use a persistent Markov chain such as PCD or PT. Finally, we discuss related GRBM studies in which GRBMs have mainly been trained using a rather small learning rate and fixing the values for the standard deviations to one. This is in contrast to our work where we emphasize the importance of using a large learning rate and optimizing the variance parameter.

Due to the structural similarity the proposed initializations and gradient restriction can also be applied to GDBMs, mcRBMs, and ssRBMs. An empirical analysis of this modification on other models would be promising future work.

The implementation of the proposed modifications and the algorithms analyzed in this work are part of the Python library PyDeep publicly available at https://github.com/MelJan/PyDeep. The natural image data set is publicly available at https://zenodo.org/record/167823

## Supporting information

S1 File S1_File.py. Python code for creating the 2D data used in the experiments.
(PY) Click here for additional data file.
