# Correction: Gaussian-binary restricted Boltzmann machines for modeling natural image statistics

**DOI:** 10.1371/journal.pone.0174289

**Published:** 2017-03-15

**Authors:** Jan Melchior, Nan Wang, Laurenz Wiskott

The following information is missing from the Funding section: The study was funded by the open access publication funds of the Ruhr-Universität Bochum.
